# Australian Sea Snake Envenoming Causes Myotoxicity and Non-Specific Systemic Symptoms - Australian Snakebite Project (ASP-24)

**DOI:** 10.3389/fphar.2022.816795

**Published:** 2022-03-21

**Authors:** Christopher I. Johnston, Theo Tasoulis, Geoffrey K. Isbister

**Affiliations:** ^1^ Clinical Toxicology Research Group, University of Newcastle, Newcastle, NSW, Australia; ^2^ National Poison Centre Network, Westmead Children’s Hospital, Sydney, NSW, Australia

**Keywords:** Australian, sea snake, envenoming, envenomation, antivenom, myotoxicity

## Abstract

**Background:** Sea snakes are venomous snakes found in the warm parts of the Indo-Pacific, including around Australia. Most sea snake envenoming causes myotoxicity, but previous Australian case reports describe neurotoxicity. We aimed to describe the epidemiology and clinical presentation of Australian sea snake envenoming and the effectiveness of antivenom.

**Methods:** Patients were recruited to the Australian Snakebite Project (ASP), an Australia-wide prospective observational study recruiting all patients with suspected or confirmed snakebite >2 years. Information about demographics, bite circumstances, species involved, clinical and laboratory features of envenoming, and treatment is collected and entered into a purpose-built database.

**Results:** Between January 2002 and August 2020, 13 patients with suspected sea snake bite were recruited to ASP, 11 were male; median age was 30 years. Bites occurred in Queensland and Western Australia. All patients were in or around, coastal waters at the time of bite. The species involved was identified in two cases (both *Hydrophis zweifeli*)*.* Local effects occurred in 9 patients: pain (5), swelling (5), bleeding (2), bruising (1). Envenoming occurred in eight patients and was characterised by non-specific systemic features (6) and myotoxicity (2). Myotoxicity was severe (peak CK 28200 and 48100 U/L) and rapid in onset (time to peak CK 13.5 and 15.1 h) in these two patients. Non-specific systemic features included nausea (6), headache (6), abdominal pain (3), and diaphoresis (2). Leukocytosis, neutrophilia, and lymphopenia occurred in both patients with myotoxicity and was evident on the first blood test. No patients developed neurotoxicity or coagulopathy. Early Seqirus antivenom therapy was associated with a lower peak creatine kinase.

**Conclusion:** While relatively rare, sea snake envenoming is associated with significant morbidity and risk of mortality. Early antivenom appears to have a role in preventing severe myotoxicity and should be a goal of therapy.

## Introduction

Sea snakes (*Hydrophiini* or true sea snakes**)** are a diverse clade of venomous Elapidae, that are part of the Hydrophiinae sub-family, which also include all Australia-Papuan/Melanesian terrestrial elapids and sea kraits (*Laticauda*) (Strickland et al., 2016; Zaher et al., 2019). True sea snakes live exclusively in water and are commonly found in the warm tropical and subtropical parts of the Indian and Pacific Oceans, where they live in shallow coastal waters, estuaries, the open ocean, and occasionally inland lakes and rivers (Phillips, 2002; Sanders et al., 2008; Lane and Shine, 2011). Until recently some sea snake species in Australian waters were thought to be the same as those found in Asia. Recent morphological and molecular evidence has demonstrated distinct lineages in Australia. An important example is the previously recognised beaked sea snake *Enhydrina schistosa* in Australia has now been made a separate species, *Hydrophis zweifeli* (Ukuwela et al., 2013; Rasmussen et al., 2014). Sea snakes are easily identifiable by their vertically flattened, paddle-shaped tail, and most species are not considered to be aggressive unless they are being handled, threatened, or it is mating season (Mcgoldrick and Marx, 1991). World-wide sea snake bites are rare, with many sea snake bites occurring from fishing activities (Marsden and Reid, 1961; Reid, 1975b; Limpis, 1978).

Sea snake venoms have been studied extensively, with numerous toxins identified and studied in animal models. Myotoxins (Fohlman and Eaker, 1977; Ali et al., 2000) and neurotoxins (pre- and post-synaptic) (Geh and Chan, 1973; Tu, 1974; Chetty et al., 2004) are the most important venom components Other toxins that have been identified include cytotoxins (Tamiya and Yagi, 2011) and haemolysins (Tu et al., 1970; Tu, 1974), although the clinical relevance of these toxins is unclear.

Most published cases of sea snake envenoming come from Malaysia (Marsden and Reid, 1961; Reid, 1975b), Sri Lanka (Kularatne et al., 2014), and Thailand (Sitprija et al., 1971), with male fisherman the highest risk group (Reid, 1975b). The reported rate of envenoming after witnessed bites was 32% (Reid, 1975b). The commonest clinical effects are systemic myotoxicity, with muscle pain and tenderness, muscle stiffness and spasm, weakness, elevated serum creatine kinase (CK), myoglobinuria, and myonecrosis on muscle biopsy (Reid, 1961a; Marsden and Reid, 1961; Kularatne et al., 2014). A small proportion of patients with myotoxicity developed an acute kidney injury with associated uraemia, severe hyperkalaemia and anuria, with some treated with renal replacement therapy (Marsden and Reid, 1961; Sitprija et al., 1971). Several deaths are reported from respiratory failure, hyperkalaemia and acute kidney injury (Marsden and Reid, 1961). In some cases, features of neurotoxicity are reported, with ptosis, ophthalmoplegia, flaccid paralysis, and loss of tendon reflexes. These neurological features were believed by the investigators to be a result of severe myotoxicity, rather than a direct neurotoxic effect (Marsden and Reid, 1961; Reid, 1975a; Kularatne et al., 2014). Other features of sea snake envenoming that were reported include leucocytosis and vomiting (Reid, 1961a; Reid, 1975a). Minimal local bite site effects were noted (Reid, 1961b).

Few sea snake envenoming cases have been reported in Australia, and the envenoming syndrome remains poorly characterised (Mercer et al., 1981; Fulde and Smith, 1984; Dobb, 1986; Patterson and Swallow, 1991). Neurotoxicity is the commonest finding reported in case reports, with slurred speech, paraesthesia, ptosis, diplopia, ophthalmoplegia, muscle weakness and flaccid paralysis, and other neurological signs (hyperreflexia, drowsiness, loss of consciousness and confusion) (Mercer et al., 1981; Fulde and Smith, 1984; Dobb, 1986; Patterson L, 1991). Myotoxicity has also been reported, as well as opisthotonos, respiratory distress, headache, vomiting, mild coagulopathy, and leucocytosis (Mercer et al., 1981; Fulde and Smith, 1984; Dobb, 1986; Patterson and Swallow, 1991; Seqirus Pty Ltd, 2019). A case of fatal sea snake envenoming in Australian waters has been reported, with rapid onset of neurotoxic features and death from respiratory failure (Tiemensma and Byard, 2021).

There is a commercial sea snake antivenom (Seqirus^®^) available for the treatment of sea snake envenoming, which is raised against Malaysian *Hydrophis schistosa* (beaked sea snake) venom in horses (Ukuwela et al., 2013; Seqirus Pty Ltd, 2019). The product is manufactured in Australia as vials to be diluted and given as an intravenous infusion (Seqirus Pty Ltd., 14th November 2019). The product requires refrigeration and has a shelf life of 3 years (Therapeutic Goods AdministrationA. G., 2020). It’s cost and cold chain storage requirements limit availability in many countries that have cases of sea snake envenoming, with some availability and usage described in Malaysia and Singapore (Tan, 2010; Tan et al., 2015). Despite being raised against only one sea snake venom to a species not found in Australian waters, the product is recommended for envenoming from all sea snake types in Australia (Seqirus Pty Ltd, 2019). Efficacy in animal models of neurotoxicity and lethality has been demonstrated with the venoms from many sea snake species (Tu, 1987; Chetty et al., 2004). The antivenom appeared to be effective in a small series of patients from Malaysia (Reid, 1962; Reid, 1975a).

The objective of this study is to examine the epidemiology and clinical presentation of Australian sea snake envenoming and investigate the effectiveness of sea snake antivenom in its treatment.

## Methods

We undertook a review of all sea snakebites recruited to the Australian snakebite project. The Australian snakebite project is a prospective multi-centre observational study of patients with suspected snakebite from all Australian states and territories. All patients with suspected or confirmed snakebite over the age of two are eligible for recruitment. Human research ethics approval was obtained from major State and Territory human research ethics committees (HRECs) responsible covering all recruiting hospitals including the Northern Territory Department of Health and Menzies School of Health Research (reference, 04/08), the Hunter New England Area Health Service and the University of Newcastle (reference, 07/11/21/3.06), the Royal Perth Hospital Ethics Committee and South Metro Area Health Service (reference, RA-08/003), the Western Australian Country Health Service (reference, 2008:03), the Tasmania Network (reference, H00109965), and the Gold Coast Health Service District (reference, 200835), as well as for a further ten HRECs of participating facilities. Written informed consent was obtained from the patient or their next of kin, parent or guardian (for minors or those unable to provide consent themselves).

Patients are referred to the ASP investigators for recruitment after identification of snakebites by onsite clinicians, local investigators, staff of the Australian Poisons Information Centre Network or laboratory staff. Patient information sheets, consent forms, data collection forms and laboratory procedures are faxed to the treating site. After the patient has provided informed consent, information is collected on demographics, bite circumstance, clinical data, treatment given and response, in purpose designed datasheets that are completed by the treating team. Missing data is collected from patient medical records if required. Patient treatment is determined by the local treating team, in many cases in consultation with a Clinical Toxicologist from the Australian Poisons Information Centre Network. Collected data are entered into a purpose designed database by trained research assistants and then reviewed by the chief investigator.

Clinical syndromes are defined based on signs and symptoms of envenoming and serial laboratory findings, according to pre-determined criteria (Johnston et al., 2017). Myotoxicity was defined as patients with a CK greater than 1000 U/L and severe myotoxicity as a CK greater than 10,000 U/L (Johnston and Isbister, 2020). Systemic hypersensitivity reactions to antivenom are defined by the National Guidelines of Allergy and Infectious Disease—Food Allergy and Anaphylaxis Network criteria (Sampson et al., 2006), with severity defined by the grading system proposed by Brown (2004).

The ASP database was searched from January 2002 to August 2020 for all potential cases of sea snakebite, including both envenomed and non-envenomed patients. Included patients were those with likely sea snakebite, defined as either: 1) a patient bitten by a sea snake in which there was expert identification of the snake involved or 2) there was witnessed bite by a snake with a paddle-shaped tail in, or nearby, coastal waters. Information extracted from the database included patient demographic data, bite circumstance and location, clinical findings, laboratory investigations, snakebite treatment and time to hospital discharge post-bite.

Descriptive statistical analysis is carried out on continuous outcomes, with normality of the data assessed by the Kolmogorov-Smirnov test and the Shapiro-Wilk normality test. All descriptive data is presented as medians with interquartile ranges (IQR) and ranges. All analyses and graphics were done in GraphPad Prism version 9.0.2 for Windows, GraphPad Software, San Diego California, United States.

## Results

Between January 2002 and August 2020 there were 2,292 patients recruited to the Australian Snakebite Project. Thirteen sea snake bites were identified, making up only 0.6% of all snakebites. Eleven of 13 were male and the median patient age was 30 years (4–68 years). One patient was a snake handler (Table 1).

**TABLE 1:** Summary of patient characteristics and clinical features of 13 patients with sea snake bite.

Age/Sex	Location	Activity	Bite site	Sea snake type	PBI used	Envenomed	Features of envenoming	Muscle pain/tenderness	Peak CK (U/L)	AV given	Time to first AV post-bite (h)	Antivenom reaction	Time to discharge post bite (h)
4/M	Beach (in water)	Swimming	Hand	Unknown	No	Yes	Myotoxicity, NSSS	General	48,100	Yes	9	No	26.0
12/M	Beach (in water)	Fishing	Ankle	Unknown	Yes	Yes	Myotoxicity	Local	28,200	Yes	11.5	No	166.0
30/M	Ocean	Fishing on trawling vessel	Finger	Unknown	Yes	Yes	NSSS	Local	939	Yes	5.5	No	35.5
15/F	Beach (in water)	Swimming	Leg	Unknown	Yes	No	Mild envenoming*	No	919	Yes	14.75	Moderate	45.0
23/M	Ocean	Fishing on trawling vessel	Finger	Unknown	Yes	Yes	NSSS	No	804	Yes	4.25	Mild	36.0
52/M	Ocean	Spear fishing	Face	Unknown	No	Yes	NSSS	General	275	Yes	4	No	22.5
51/M	Ocean	Collecting research specimens	Finger	*Hydrophis zweifeli*	Yes	Yes	NSSS	No	146	No	N/A	No	18.8
17/F	Beach (in water)	Walking in shallow water	Foot	Unknown	Yes	Yes	NSSS	Local	86	Yes	4.75	No	16.9
30/M	Ocean	Fishing on trawling vessel	Finger	Unknown	No	No	Non-envenomed	No	Not available	No	N/A	No	14.3
16/M	River bank	Fishing	Finger	*Hydrophis zweifeli*	Yes	No	Non-envenomed	No	223	No	N/A	No	19.5
62/M	Beach (on sand)	Fishing	Finger	Unknown	Yes	No	Non-envenomed	No	210	No	N/A	No	9.0
68/M	Ocean	Fishing on trawling vessel	Finger	Unknown	Yes	No	Non-envenomed	No	203	Yes	6.5	No	22.0
47/M	Beach (on sand)	Recovering unwell snake	Finger	Unknown	Yes	No	Non-envenomed	No	58	No	N/A	No	11.5

NSSS, Non-specific systemic symptoms. *—Mild envenoming with rapid rise in CK (but peak less than 1000 U/L thus not meeting definition of myotoxicity).

**FIGURE 1:**
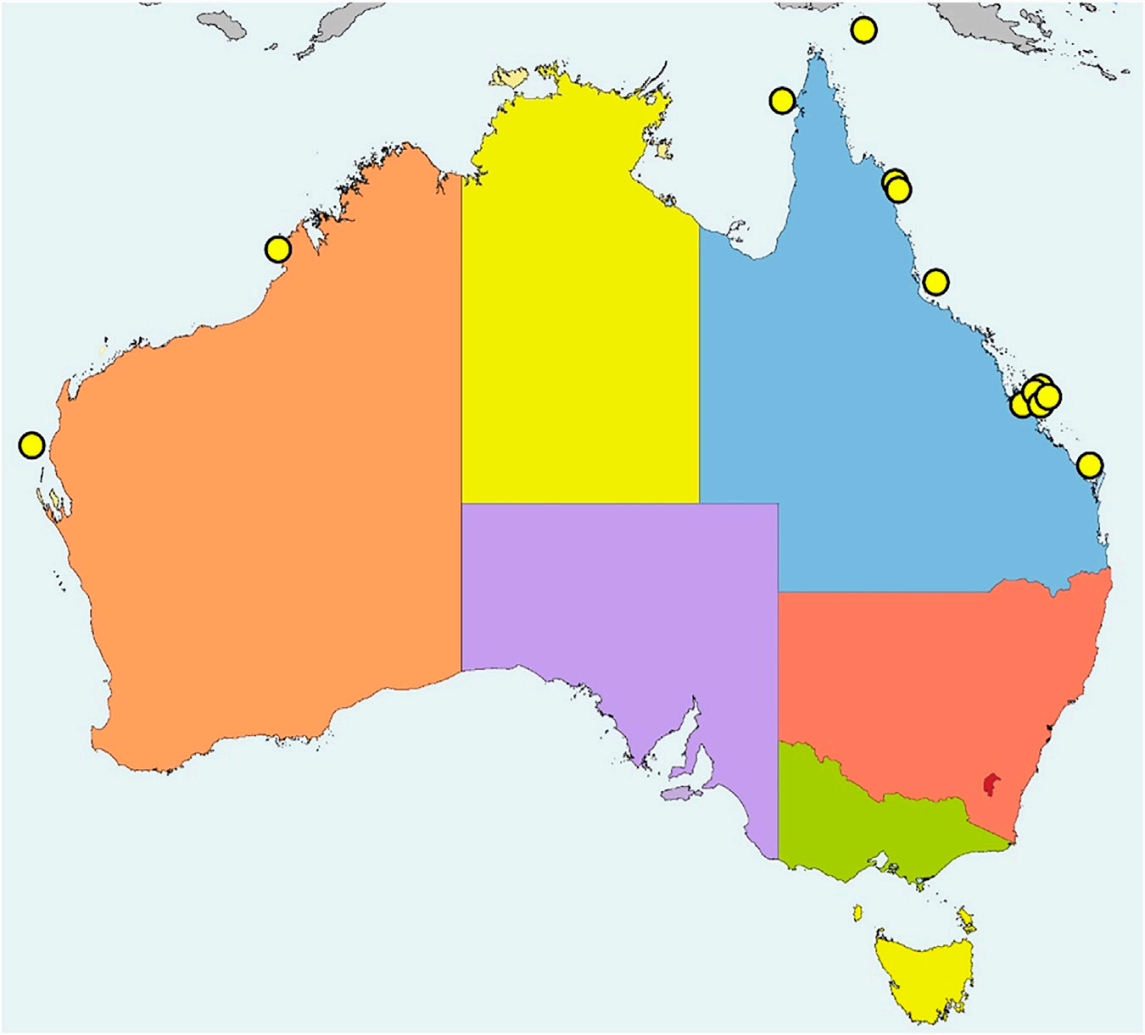
Distribution of 13 sea snake bites in Australia from January 2002 to August 2020.

**FIGURE 2:**
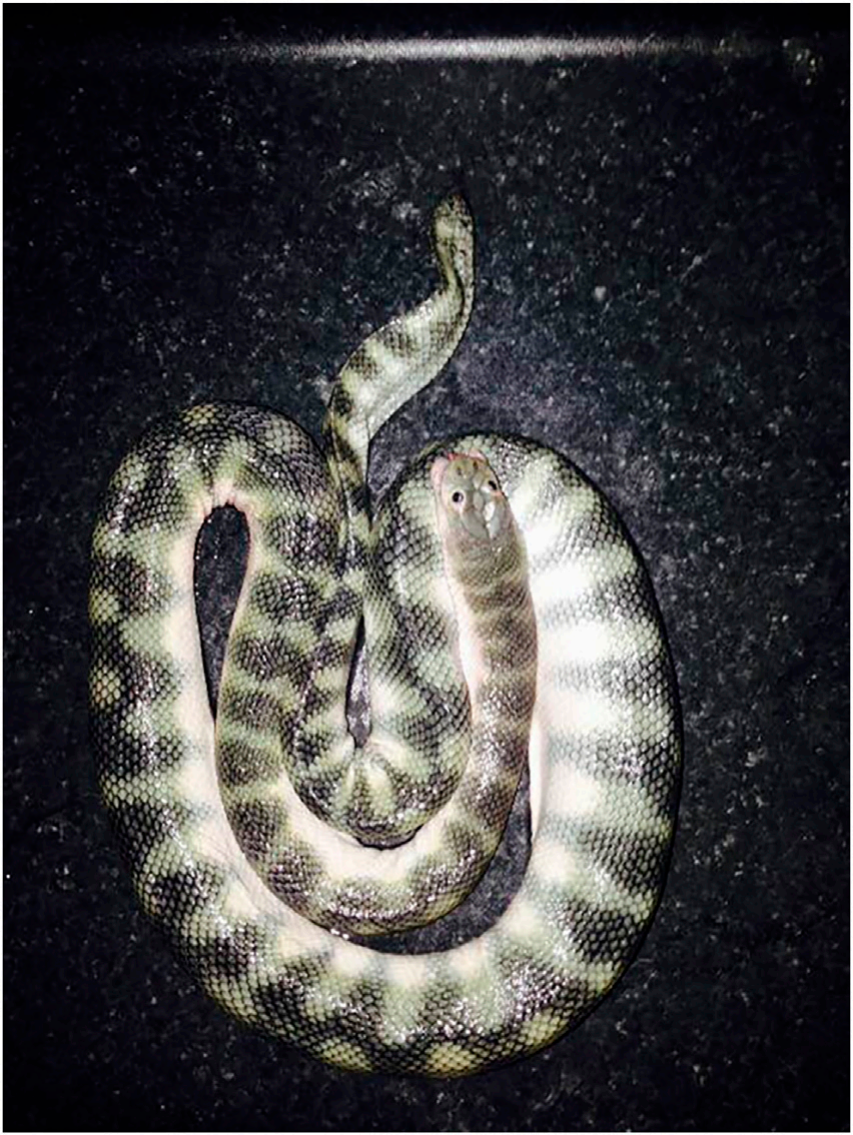
Photograph of a *Hydrophis zweifeli* (Beaked sea snake—formerly known as Australian *Enhydrina schistosa* ) (credit: Jamie Seymour).

All bites occurred in the wild on the coast of, or waters off, Queensland and Western Australia, of which the most southern location was Hervey Bay, Queensland (Figure 1). All patients were in or near the water at the time of the bite: four on boats, four swimming in the ocean, three standing on land (two on the beach, one on a river bank) and two standing in shallow water. The only inland bite occurred on the bank of the Fitzroy river at Rockhampton (approximately 56 km inland) (Figure 1). Activity at the time of bite included fishing (11), swimming (2), standing on the snake (1), handling a snake found on land (1) and collecting marine animals for research (1). There were nine patients bitten on the upper limb, three on the lower limb and one patient who was bitten on the face (Table 1). There was identification of the snake species involved in two cases, both *Hydrophis zweifeli* (Beaked sea snake—formerly known as Australian *Enhydrina schistosa*; Figure 2).

**FIGURE 3:**
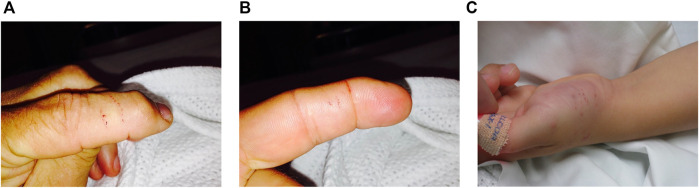
(**A–C)**: Fang marks of patients bitten by sea snakes.

**FIGURE 4:**
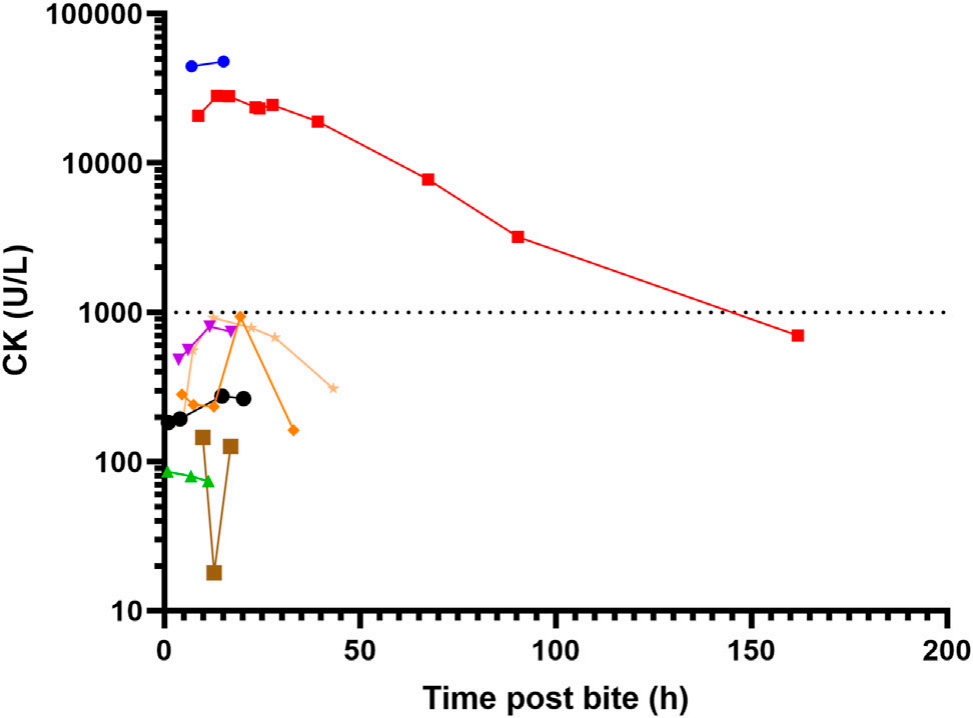
Serial creatine kinase (CK) measurement for eight patients with sea snake envenoming, two with myotoxicity (peak CK > 1000 U/L).

Local effects in 9 patients included pain (5) that was only mild, minimal swelling (5) local bleeding (2) and bruising (1) (Figure 3). Systemic envenoming occurred in eight of the 13 patients and was characterised by non-specific systemic symptoms (6) and myotoxicity (2). In addition to those with myotoxicity four further envenomed patients had small elevations in creatine kinase (CK) of 275 U/L, 804 U/L, 919 U/L, and 939 U/L (Table 1; Figure 4). Non-specific systemic symptoms included nausea (6), headache (6), abdominal pain (3), and diaphoresis (2). Median time to onset of first non-specific systemic symptoms was 22.5 min (IQR: 5–119 min, range 5–385 min). Five envenomed patients had musculoskeletal symptoms, including local myalgia (4), local muscle tenderness (2), generalised myalgia (2), and trismus (1). Median time to onset of first musculoskeletal symptoms was 30 min (IQR: 12.5–195 min; range 10–210 min). Two patients had myotoxicity with a peak CK of 48,100 U/L and 28,200 U/L, and therefore severe myotoxicity. Neither developed an acute kidney injury. The time to peak CK was 15.1 and 13.5 h, respectively in these two patients (Figure 4). They had elevated serum transaminases, peak aspartate transaminase (AST) of 2350 U/L and 748 U/L, and peak alanine transaminase (ALT) of 514 and 241 U/L, respectively. Both patients with myotoxicity also had an elevated white cell count (WCC of 14.2 × 10^9^/L and 15.7 × 10^9^/L), evident on the first blood test (7 and 8.6 h). Both had neutrophilia and lymphopenia. Serum AST and ALT results were available in five of the other patients, respectively, and were within normal limits in all of these patients. Nine of the eleven other patients had WCC results and only one patient (who was non-envenomed) had an elevated WCC of 11.6 × 10^9^/L. No patients had objective evidence of neurotoxicity, venom-induced consumption coagulopathy, anticoagulant coagulopathy, thrombocytopenia, haemolysis, hyperkalaemia, or acute kidney injury.

Pressure bandage with immobilisation was performed in ten of the patients. Antivenom was given to seven of the eight envenomed patients, and one non-envenomed patient. All patients initially received one vial of antivenom, and only one patient received a second vial. The median time to the first dose of antivenom post-bite was 6 h (IQR: 4.4–18.9; range 4–14.8 h). Administration of antivenom within 6 h in patients with envenoming appeared to be associated with lower peak CK in envenomed patients, with a peak CK measurements of 919, 28,200, and 48,100 U/L for the ≥ 6-h group, versus 86, 275, 804, and 939 U/L for the < 6-h group. Two patients had immediate hypersensitivity reactions to antivenom. The first had a mild hypersensitivity reaction limited to urticaria managed with an antihistamine and adrenaline infusion. The second developed a moderate hypersensitivity reaction with cough and angioedema, managed with an antihistamine and corticosteroid.

All patients survived to discharge and the median time to discharge post-bite for the 13 patients was 22 h (IQR: 15.6–35.8; range 9–166 h) (Table 1). Time to discharge for the two patients with myotoxicity were 26 and 166 h post-bite, respectively (Table 1).

## Discussion

Australian sea snake bites are extremely rare, with less than one case per year on average, and only half of these envenomed. We found that sea snake envenoming in Australia is characterised by non-specific systemic symptoms and myotoxicity, the latter is both rapid in onset and severe. Neurotoxicity was not observed in this case series, consistent with the international literature (Marsden and Reid, 1961; Sitprija et al., 1971; Kularatne et al., 2014). Laboratory abnormalities included increased CK consistent with muscle injury, elevated AST and to a lesser extent ALT, and neutrophilia and lymphopenia. The elevated AST and ALT are more likely to be due to muscle injury, rather than liver injury, because the more specific liver enzyme ALT was less elevated. Venom induced consumption coagulopathy and anticoagulant coagulopathy did not occur, in contrast to most important Australian terrestrial snakes (Johnston et al., 2017). Administration of antivenom within 6 h appeared to be associated with a reduction in the severity of myotoxicity.

The relative rarity is surprising, given the wide distribution of several sea snake species in Australian waters and the marked potential for occupational exposure, particularly with the prawn trawling activity off the north coast of Australia, with tens of thousands of sea snakes caught in fishing nets per year (Wassenberg et al., 1994). More importantly, the rarity of sea snakebites makes it more difficult to have antivenom available for patients, which was reflected in the longer times to antivenom administration compared to terrestrial snake envenoming (Johnston et al., 2017).

This case series lacked fatal cases of sea snake envenoming, previously seen particularly in published research from Malaysia (Marsden and Reid, 1961), and described previously in Australia (Tiemensma and Byard, 2021). There are a number of potential reasons for this, including variations involving the snake (snake type, regional variation in snake venom toxicity, age of the snake), bite circumstances (part of body affected, amount of venom injected), patient (age, weight and comorbidities), and treatment (first aid administered and time to access of hospital care, investigations, antivenom, and supportive care). There were no cases of sea snake envenoming identified post-mortem in a previous series including coronial cases of snake envenoming (Johnston et al., 2017). This may be due to the relative rarity of sea snake envenoming in Australia and the quality of care, as well as the difficulty conclusively diagnosing sea snake envenoming post-mortem (Feola et al., 2020).

While only present in two patients, the myotoxicity that occurred was striking in both the speed of onset and severity. Both patients had markedly elevated CK on first blood test results at 7 and 8 h respectively, which was different to myotoxicity reported from Australian terrestrial snakes, in which the median time to first abnormal CK was 11.1 h and the median time to peak CK was 34.3 h (Johnston and Isbister, 2020). For the one patient with serial CK results available, the CK peaked early at 13.5 h. Unfortunately, the other patient was discharged after the second CK result of 48,100 U/L, because he was clinically well (Figure 4). The speed of onset is in keeping with several fatal cases caused by severe myotoxicity described by Marsden and Reid in Malaysia, in which death occurred less than 24 h post-bite (Marsden and Reid, 1961). Some previously described musculoskeletal features of envenoming, such as severe muscle spasms, were not observed in these patients. Isolated elevation in serum transaminases is a previously described phenomenon in Australian snakebite associated myotoxicity (Johnston and Isbister, 2020), and likely relates to release of release of transaminases from damaged skeletal muscle, rather than primary hepatotoxic effect.

Neurotoxicity, a feature previously described in Australian cases of sea snake envenoming, including one fatal case, did not occur in this series (Tiemensma and Byard, 2021). Even though several neurotoxins have been identified in sea snake venoms, they are of unclear clinical relevance in human envenoming, or there is known inter-species variation in response to neurotoxins, as is the case with the lack of binding site for short chain neurotoxins from *H. schistosa* venom in humans (Harris, 1989). In a Malaysian case series of sea snake envenoming fatalities, early mortality from issues such as paresis and respiratory failure is described, features often attributed to neurotoxicity (Marsden and Reid, 1961). Subsequent autopsy on some cases in the Malaysian series demonstrated marked necrosis of intercostal and diaphragmatic muscle, with myotoxicity, rather than neurotoxicity, attributed as the cause of death (Marsden and Reid, 1961). In a small number of sea snake envenoming cases from Sri Lanka (Kularatne et al., 2014) and Thailand (Sitprija et al., 1971) neuromuscular transmission and nerve conduction studies have been carried out, demonstrating myopathy as the cause of neurological symptoms in the patients. The authors of the Thai case series also proposed that myotoxic sensitisation of skeletal muscle could potentiate the paralytic effects of hyperkalaemia and uraemia, with rapid improvement in symptoms noted when patients were treated with haemodialysis with associated correction of serum potassium and urea excess (Sitprija et al., 1971). However, neurotoxicity may occur in only some species of sea snakes, which may not have caused bites in this series.

To date, there is minimal clinical evidence published to support the recommended treatment for Australian sea snake envenoming, Seqirus sea snake antivenom^®^. Potential limitations of the treatment include that it is monovalent and raised against venom from *Enhydrina schistosa* (beaked sea snakes) of Malaysian origin, especially given recent recognition of Australian *E. schistosa* actually being a distinct species, *Hydrophis zweifeli* (Reid, 1961b; Ukuwela et al., 2013). Despite this, the antivenom is recommended for all envenomed cases from a diverse local sea snake fauna (Seqirus Pty Ltd, 2019). Whilst *in vitro* and animal modelling of antivenom efficacy has been demonstrated, it is unclear if the models chosen or inter-species variability in response translates into effectiveness in human cases for all sea snake types. In the absence of being able to confirm specific sea snake type and quantify effectiveness in a larger group of patients, this study demonstrates the potential value of early antivenom in preventing severe myotoxicity. The use of tiger snake antivenom for the management of sea snake envenoming, a practice that was previously recommended if sea snake antivenom was not available (Acott, 1986) prior to antivenom manufacturing changes (Chetty et al., 2004), was not observed and is not recommended.

There are several limitations to this study, the most important of which is the lack of venom-specific enzyme immunoassay, which can allow identification of snake species involved, quantify venom present, allow correlation of venom concentration with clinical presentation, and measure the efficacy of sea snake antivenom at binding circulating venom. Given the diverse sea snake fauna in Australia, it is difficult to obtain venom samples from this broad range of difficult to catch snakes and prepare polyclonal antibodies for all potential snakes. This, coupled with snake specimens or photo availability, led to identification of the involved sea snake being possible in only two cases. The generalisability of observational data (some of which is secondary data, such as physical examination and clinical features that are not directly collected by the research team) in the small number of cases described in this series to sea snake bite from any species in Australia is also not known.

Despite the rarity of sea snake bite in Australia, half of the patients were envenomed and envenoming appears to be associated with significant morbidity and potentially mortality when it occurs. Whilst difficult to definitively diagnose without the presence of coagulopathy, patients with potential sea snakebite based on appearance of the snake and locality, should have close serial physical examination and laboratory testing. Strong consideration of early antivenom therapy (<6 h) should be given for patients with non-specific systemic features, muscle pain and tenderness, or neutrophilia, with a goal of preventing fulminant myotoxicity from developing.

## Data Availability

The raw data supporting the conclusion of this article will be made available by the authors, without undue reservation.
